# High prevalence of diabetic retinopathy and lack of association with integrin α2 gene polymorphisms in patients with type 2 diabetes from Northeastern Mexico

**DOI:** 10.3892/etm.2015.2520

**Published:** 2015-05-26

**Authors:** ANA CECILIA CEPEDA-NIETO, MARÍA TERESA ESQUIVEL-CONTRERAS, FRANCISCO DURAN-IÑIGUEZ, MAURICIO ANDRÉS SALINAS-SANTANDER, HUGO LEONID GALLARDO-BLANCO, SANDRA CECILIA ESPARZA-GONZÁLEZ, ALEJANDRO ZUGASTI-CRUZ, JESÚS ANTONIO MORLETT-CHÁVEZ, LUIS TLALOC CÓRDOVA-ALVELAIS

**Affiliations:** 1 Research Department, Faculty of Medicine, Autonomous University of Coahuila, Saltillo, Coahuila 25000, Mexico; 2 Faculty of Chemical Sciences, Autonomous University of Coahuila, Saltillo, Coahuila 25280, Mexico; 3 Department of Biochemistry and Molecular Medicine, Faculty of Medicine, Autonomous University of Nuevo León, Monterrey, Nuevo León 64460, Mexico

**Keywords:** α2 integrin gene, polymorphism, diabetic retinopathy, Mexican population

## Abstract

Diabetic retinopathy (DR) is one of the primary causes of blindness in the working age population and is characterized by angiogenesis in the retina. Platelets have been suggested to be involved in the pathogenesis of diabetic microvascular complications. The integrin receptor for collagen/laminin, α2β1, mediates platelet primary adhesion to subendothelial tissues, which is an essential first step in thrombus formation. The gene encoding the α2 subunit of α2β1 integrin has ≥8 polymorphisms, including a *Bgl*II/*Nde*I restriction fragment length polymorphism. To explore the prevalence of DR in a population from Northeastern Mexico, unrelated, hospitalized patients who had received a diagnosis of type 2 diabetes mellitus (DM2) at least 10 years previously were recruited (n=177). DR was diagnosed in a masked manner by independent ophthalmologists using fundus images captured using a non-mydriatic retinal camera. A total of 121 patients with DM2 (68%) had some degree of DR development (DR patients), and 56 patients with DM2 (32%) did not exhibit any sign of DR (No-DR patients). The results showed that after 15 years of DM2 progression, there is an increased risk of DR (P=0.0497; odds ratio, 1.993). In addition, insulin therapy and family history of DM2 were significantly associated with DR. In order to detect a possible association between DR and *Bgl*II/*Nde*I α2 gene polymorphisms, a comparative cross-sectional study between DR and No-DR patients was conducted. The α2 gene was genotyped by polymerase chain reaction-restriction fragment length polymorphism assay. Statistical analysis revealed no association between *Bgl*II/*Nde*I genotypes and the development of DR in this group of patients. In conclusion, the present data indicate a high prevalence of DR in the Mexican population and suggest that the damage in DR is due to other factors, such as the duration of the DM2, and is not linked to *Bgl*II/*Nde*I α2 gene polymorphisms.

## Introduction

The most common complication of type 2 diabetes mellitus (DM2) is diabetic retinopathy (DR), which is a microvascular complication of the retina and remains one of the leading causes of blindness throughout the world (1). The characteristic features of DR include increased vascular permeability and hemostatic abnormalities, which may result in vascular occlusion, irrespective of antidiabetic treatment, and, ultimately, retinal non-perfusion and neovascularization (2). Platelets have been suggested be involved in the pathogenesis of DR (3,4).

DR can be classified into early- and late-stage categories. The early stage of DR, also known as non-proliferative diabetic retinopathy (NPDR), is associated with edema, fluid leakage and restricted blood flow into the eye, in the absence of abnormal neovascularization. By contrast, advanced-stage DR, or proliferative diabetic retinopathy (PDR), is characterized by neovascularization and fibrous tissue formation (5).

Population-based studies in different countries have found the prevalence of DR in individuals diagnosed or undiagnosed with DM2 to range from 17.6% in India to 54% in Iran (6–8); however, these data are scarce, as these studies rely on sophisticated diagnostic equipment (7–14). The reported prevalence rate of DR in a Mexican-American population aged ≥40 years was found to be 48% (15,16). In Mexico, a study reported 38.9% DR prevalence in a population from Southern Mexico (17). The high prevalence in Southern Mexico is consistent with data from another study undertaken in Mexico, in which the DR prevalence was 51% (18).

In Coahuila, Mexico, the number of patients with DM2 has increased over recent years (19); therefore, the number of patients with DR is also increasing. To date, there are no recent reports concerning DR prevalence in Northeastern Mexico, and thus investigating the DR prevalence in this region is one of the purposes of this research.

Evidence from ethnic and family studies has suggested that there may be genetic susceptibility for DR. In the Diabetes Control and Complications Trial, a 3.1-fold increased risk of severe retinopathy was found for subjects with retinopathy-positive relatives, and a correlation for the severity of retinopathy was present among all family members (20). Similar results for the clustering of DR-positive individuals within families were also observed among Southern Indians and Mexican Americans with DM2 (21,22). In the FIND-Eye study, the overall broad-sense heritability for DR was found to be 27%, compared with 24% in Mexican-American families (23). The role of genetics in DR is believed to be responsible for the fact that certain diabetic individuals avoid retinal complications, even following a long history of uncontrolled diabetes, while others rapidly develop DR, despite tight metabolic control (24).

Considerable research has been performed to identify the genetic markers that are associated with the risk of developing DR using an indirect approach, such as case-control association studies, in a number of ethnic populations. In these studies several candidate genes with different functions have been reported to be associated with DR (3,25–33); however, only a fraction of these studies have shown a consistent association with DR or its severity in different populations (25,34–37).

The pathogenesis of DR is linked with vascular permeability, tissue ischemia and angiogenesis (5). Alterations in platelet function are frequent in patients with DM2 and contribute to the pathogenesis and progression of vascular complications (3,14,38,39). Following vascular injury, circulating blood platelets are exposed to subendothelial collagen, a protein that stimulates the matrix adhesion and activation required for platelet thrombus formation. The firm adhesion of platelets to collagen requires the involvement of collagen receptors, such as integrin α2β1 (38,40). Given the important role of integrin receptors in vascular processes and their complications, these receptors are considered a good candidate for investigations into a possible association with the DR in patients with DM2.

Integrin receptors are heterodimeric molecules composed of non-covalently associated α and β subunits that mediate cell-cell and cell-matrix adhesion (38,40). α2β1, also known as the platelet membrane glycoprotein Ia-IIa complex or ITGA2, is a membrane glycoprotein expressed in megakaryocytes and blood platelets. This receptor acts as a platelet receptor for collagen (38,40) and mediates the primary adhesion of platelets to subendothelial tissues, which is a crucial first step in the formation of thrombi. The expression levels of the integrin α2β1 in platelets vary significantly among normal individuals, whereas the levels of other integrin receptors do not (40,41).

The gene encoding the α2 subunit of α2β1 integrin has ≥8 polymorphisms, including two conservative changes in the amino acid coding region of α2 at nucleotides 807 (TTT/TTC at codon Phe^224^) and 873 (ACA/ACG at codon Thr^246^) of the cDNA sequence (41). These DNA sequence polymorphisms, referred to as 807C and 807T for the 807C/873G and 807T/873A pairs, respectively, in the α2 gene coding region are linked with two polymorphic sites in the α2 gene intron G (3,160 A/G and 3,090 T/C), which correspond to the recognized sites by *Bgl*II and *Nde*I restriction enzymes, respectively (41).

The *Bgl*II and *Nde*I recognition sites in intron G of the α2 gene are used to genotype three different α2 gene alleles defined by eight nucleotide polymorphisms, including the 807C and 807T polymorphisms, using *Bgl*II/*Nde*I restriction analysis (41). The allele *Bgl*II (+)/*Nde*I (+) has been linked to 807T polymorphism, which is associated with high levels of platelet integrin α2β1 expression, while allele *Bgl*II (-)/*Nde*I (-) has been linked to 807C polymorphism and is associated with low levels of platelet integrin α2β1 (3,4,41).

Associations between the *Bgl*II polymorphism and the prevalence of DR have been reported in Japanese (3) and Caucasian (25) diabetic patients, and the *Bgl*II (+) allele in these two ethnic groups has been shown to be a risk factor for DR; however, the association between the *Bgl*II/*Nde*I polymorphism and the prevalence of DR has not yet been studied in patients with DM2 from Northeastern Mexico. The aim of the present study, therefore, was to test the hypothesis that a genetic variation in the α2 integrin gene is associated with the development of DR by analyzing the possible association between the intronic polymorphic sites *Bgl*II and *Nde*I of the α2 gene and the susceptibility to DR among Mexican diabetic patients with ≥10 years of DM2 history.

## Materials and methods

### 

#### Study subjects

A comparative cross-sectional hospital study was performed to analyze the clinical characteristics of DR and to explore the genetic association between DR and polymorphic variants of the α2 gene. Unrelated Mexican patients who had been diagnosed with DM2 ≥10 years previously (n=177; 108 males, 69 females) were recruited at the Mexican Social Security Institute (IMSS) Hospital (Saltillo, Mexico). Diabetes was defined according to the report of the expert committee on the diagnosis and classification of diabetes mellitus (42). Only DM2 patients were included in this study, and the study was performed in accordance with the ethics standards of the Dr Gonzalo Valdés University Hospital of the Autonomous University of Coahuila (Saltillo, Mexico). The University Hospital-Autonomous University of Coahuila Institutional Review Board approved and registered the study under the code FM001-10.

Informed consent was obtained from all subjects enrolled following an explanation of the nature of the study. Patients with known DM2 for a duration of <10 years, patients who had not voluntarily agreed to participate in the study and those who did not have a laboratory workup were excluded from the study.

#### Clinical data collection and study group classification

DR was diagnosed in a masked manner by independent ophthalmologists using fundus images captured using a Canon CR-DGi non-mydriatic retinal camera with the Eye Q Prime software (Canon USA, Inc., Lake Success, NY, USA) in patients with a DM2 duration of ≥10 years. Patients with DM2 but without DR (No-DR group, n=56) were compared with DM2 patients with DR of either the NPDR or PDR subtype (DR group, n=121) according to Early Treatment Diabetic Retinopathy Study (ETDRS) criteria (5).

The following clinical and biochemical characteristics were analyzed for all patients with DM2 (No-DR and DR groups): Age (years), gender, duration of diabetes (years), systolic and diastolic pressure (mmHg), fasting plasma glucose (mg/dl), cholesterol (mg/dl), triglycerides (mg/dl), cataracts, glaucoma and insulin use. Documented information also included family history of DM, hypertension, cardiovascular disease, dyslipidemia and renal failure. Peripheral blood was obtained from all patients with DM2 following a 12-h fast for biochemical analysis.

#### DNA extraction

Peripheral venous blood samples (5 ml) were obtained from the DR and No-DR patients and were collected in EDTA tubes (BD Vacutainer®; Becton Dickinson, Mexico City, Mexico). The samples were centrifuged using a refrigerated centrifuge (Thermo Fisher Scientific, Inc., Waltham, MA, USA), and the buffy coat was processed for high-molecular weight genomic DNA isolation by the salting-out method, and suspended in Tris-EDTA (pH 7.8; Mallinckrodt Baker, Inc., Phillipsburg, NJ, USA), at a final concentration of 0.1–1.0 µg/µl.

#### Genotyping for the α2 gene 3,160 A/G and 3,090 T/C polymorphisms

Genotyping for the α2 gene was performed to analyze the possible genetic association between DR and the polymorphic variants of the α2 gene. The 3,160 A/G and 3,090 T/C polymorphism of the α2 intron G region were tested. The α2 gene was genotyped using polymerase chain reaction-restriction fragment length polymorphism (PCR-RFLP) analysis, as previously reported (41), using an MJ Mini™ Gradient Thermal Cycler (Bio-Rad, Hercules, CA, USA), followed by *Bgl*II and *Nde*I [New England Biolabs (NEB), Ipswich, MA, USA] restriction enzyme analysis. In brief, the method for the PCR was as follows: 250 ng genomic DNA, 0.5 µM primers (forward, 5′-GAT TTA ACT TTC CCG ACT GCC TTC-3′ and reverse, 5′-CAT AGG TTT TTG GGG AAC AGG TGG-3′), 0.2 mM deoxyribonucleotide triphosphates (Invitrogen Life Technologies, Carlsbad, CA, USA), 1.5 mM MgCl_2_ and 2.5 units of Taq DNA polymerase (Invitrogen Life Technologies) were combined to form the reaction mixture. The 600-bp segment of intron G encompassing the *Bgl*II and *Nde*I sites was amplified from genomic DNA in a final volume of 25 µl using the following conditions: 2 cycles of 94°C for 1 min, 69°C for 1 min and 72°C for 1 min; 2 cycles of 94°C for 1 min, 67°C for 1 min and 72°C for 1 min; and 30 cycles of 94°C for 1 min, 65°C for 1 min and 72°C for 1 min. The PCR samples (∼1 µg) were digested overnight with *Bgl*II and *Nde*I (NEB) at 37°C in a temperature-controlled water bath (Bio-Rad). The digested amplicons were analyzed by electrophoresis in 2% agarose gels using ethidium bromide (Invitrogen Life Technologies) and a 2UV™ transilluminator (UVP, LLC, Upland, CA, USA).

The 3,160 A allele, *Bgl*II (+), and the 3,090 T allele, *Nde*I (+), of the α2 gene result in the gain of restriction sites for the enzymes *Bgl*II and *Nde*I, respectively (41). When the 3,160 A allele or and 3,090 T allele were present, the 600-bp PCR product was digested with *Bgl*II or *Nde*I enzyme, respectively, generating two fragments of 400 and 200 bp (41). The 3,160 G allele, *Bgl*II (-), and the 3,090 C allele, Nde I (-), were detected by an absence of cuts by the *Bgl*II or *Nde*I enzyme, respectively, leaving the 600-bp PCR fragment intact.

#### Statistical analysis

All collected data were organized in a database in preparation for statistical calculations. Quantitative variables (age, systolic and diastolic pressure, total cholesterol levels, triglyceride and fasting plasma glucose levels) were compared between the No-DR and DR patients with DM2 using a Student's t-test, and the results are presented as the mean ± standard deviation. The risk factors for DR were analyzed using the GraphPad Prism version 4.0 software for Windows (GraphPad Software, Inc., San Diego, CA, USA). Fisher's exact tests were used for univariate analysis to compare the clinical and biochemical characteristics, as well as the family history data, between No-DR and DR diabetic patients. The estimated odds ratio (OR) was assessed by two-sided 95% confidence intervals (CI). The difference was considered statistically significant when P≤0.05.

The polymorphism statistical analyses were performed using the SNP and Variation Suite version 8.1.5 (Golden Helix, Inc., Bozeman, MT, USA) and the Epi Info™ 7 statistical program (USD Inc., Stone Mountain, GA, USA). Analyses of allele and genotype frequency counts were performed using the χ^2^ test. For the validation of the genotypes, the Hardy-Weinberg equilibrium (HWE) was analyzed using a Fisher's exact test (significant deviation from HWE at P>0.05).

To analyze the association between the DR genotypes and the clinical, biochemical and family history data of the DM2 patients, multiple logistic regression analysis was performed to fit the binary response, using regression coefficients for continuous predictors and indicator coefficients for categorical predictors. Association studies were performed by genotypic tests with correlation to assess the ORs, 95% CI and P-value. The Bonferroni P-value and false discovery rate (FDR) were calculated to exclude spurious associations. An OR (95% CI) >1 and a P-value P≤0.05 were considered significant.

## Results

### 

#### Clinical characteristics of the study population

The aim of this study was to explore the prevalence of DR and a possible association between DR and two α2 gene polymorphisms. The analyzed population comprised clinically diagnosed DM2 patients who attended the IMSS Hospital (Northwestern Mexico). Patients that had been diagnosed with DM2 ≥10 years previously (n=177; 108 males, 69 females) were selected for the study and examined by a certified ophthalmologist.

Fundus images were captured using a Canon CR-DGi non-mydriatic retinal camera using the Eye Q Prime software (Canon USA, Inc.). All images were analyzed and the DR progress was determined. A total of 121 patients with DM2 had some degree of DR development (DR group, 68%) and 56 patients with DM2 did not exhibit any sign of DR (No-DR group, 32%). Among the patients with DM2 who developed DR, 41.32% had NPDR and 58.67% had PDR, according to the ETDRS criteria (5).

The baseline characteristics of the 177 patients with DM2 are summarized in Table I. No significant difference between the No-DR and DR groups was found for the variables analyzed (age, cholesterol, triglycerides, glucose, systolic/diastolic blood pressure). The majority of the patients were male (DR group, 60%; No-DR group, 62%), and no significant difference in gender was found between the No-DR and DR groups (Table II). The age of the patients ranged from 28 to 89 years, with a median age of 59.68 years. More than half (85%) of the participants had an age of >50 years. The majority of the No-DR (87.5%) and DR (85.71%) patients were in the >50 years age group (Table II).

The majority of studies on DR indicate the importance of the duration of the diabetes when considering a higher incidence and progression of DR (26,43–45). In the present study it was found that there was an increased risk of DR in cases of DM2 with a duration of ≥15 years (OR, 1.993; 95% CI, 1.028–3.863; P=0.0497). Other clinical characteristics, such as hypertension, glaucoma, cataract, renal disease, dyslipidemia and glycemic control, showed no association with the presence of DR in the analyzed patients (P>0.05) (Table II); however, a significant association was found between the use of insulin therapy and the presence of DR (P=0.0290) with an OR value of 0.4026 (95% CI, 0.1793–0.9040) (Table II).

Regarding the family history of the patients, a significant association between DR and a family history of DM2 was found (P=0.0291), with an OR value of 0.4561 (95% CI, 0.2280–0.9127) (Table III). Other variables in the family history were not associated with the presence of DR (P>0.05) (Table III).

#### Genotypes and DR susceptibility

The alleles of the α2 gene in Mexican patients with DM2 were identified using the *Bgl*II/*Nde*I sites with RFLP assays. The statistical results obtained from the PCR-RFLP studies are shown in Table IV. With both polymorphisms, the heterozygous genotypes were the most prevalent in the DR [*Bgl*II (A/G), 74.4%; *Nde*I (T/C), 94.2%] and No-DR [*Bgl*II (A/G), 62.5%; *Nde*I (T/C), 96.4%] patients. Homozygous genotypes for *Bgl*III (+) and *Nde*I (+) were not found in the DM2 population tested. Statistical analysis revealed no association between heterozygous *Bgl*II/*Nde*I genotypes or *Bgl*II/*Nde*I-negative genotypes and the development of DR in this patient group (P>0.05) (Table IV).

In order to assess the HWE, the data were analyzed using the Fisher's exact test. For *Bgl*II, the P-values in the No-DR and DR groups were 0.000423 and 1.204×10^−12^, respectively; for *Nde*I, the P-values in the No-DR and DR groups were 1.080×10^−13^ and 7.966×10^−27^, respectively. In all HWE analyses, the P-value was <0.05, indicating that the genotypes were not in HWE in the No-DR and DR patients.

#### Genetic polymorphisms and family history of diseases

In addition to the clinical data, a family history of the DM2-related conditions showed no statistically significant associations with the genotypes obtained by the RFLP assays (P>0.05) (Table V). In the initial analyses, a significant association with DR was observed for family history of cardiovascular disease (*Bgl*II AG genotype: OR, 2.14; 95% CI, 1.04–4.40; P=0.037) and hypertension (*Bgl*II GG genotype: OR, 2.39; 95% CI, 1.08–5.29; P=0.029); however, the significance of the association between these genotypes and cardiovascular disease susceptibility or hypertension was lost following the application of the Bonferroni correction and FDR (P>0.05) (Table V).

## Discussion

DM2 is a growing health problem in Mexico and represents a significant cause of mortality and complications such as diabetic foot, nephropathy and retinopathy. An increase in the number of diabetic patients has been predicted; therefore, it is expected that the incidence of patients with DR will show a corresponding increase (19).

The present study comprised a comparative cross-sectional hospital study in Northeastern Mexico with patients who had a history of DM2 of ≥10 years. The patients with DM2 were clinically examined by an ophthalmologist to determine if they had DR. The prevalence of DR in the analyzed Mexican population with DM2 was 68%. To date, there have been no reports of a prevalence of DR as high as that found in this study. A high prevalence (DR 48%) has been reported in a Mexican-American population aged ≥40 years living in Arizona (15) and Latinos living in California (16). The prevalence of DR found in the present study is higher than the last records found for a Mexican population, which gave prevalences of 38.9% (17) and 51% (18); however, this coincides with the exponential increase in obesity and DM2 in the country.

Population-based studies have been conducted in several countries using photographic evidence of DR. These studies have reported a DR prevalence of 10–20% in France (9), 22% in Australia (10) and Barbados (46), 27% in Fiji (11), 54% in Iran (7), 37% in China (47), 11% in India (48) and 24.8% in the USA (12).

Although the mechanisms underlying the development of DR have yet to be elucidated, numerous risk factors have been described, including poor glycemic control, longer diabetes duration, hypertension, hyperlipidemia and albuminuria (45,49). Previous studies have found a significant correlation between elevated serum cholesterol/triglycerides and the risk and severity of DR (50,51). By contrast, other studies have shown an absence of significant correlation between elevated serum cholesterol/triglycerides and the risk of DR (25,52,53). In the present study it was found that although the levels of glucose, cholesterol and triglyceride in the patients with DM2 were above levels considered normal, these abnormal results appeared to have no association with the development of DR (P>0.05) (Table I). Hyperglycemia was observed in 73.55% of patients in the DR group and 78.57% of patients in the No-DR group (P=0.5758) (Table II). Compared with values reported in other studies (54–57), the prevalence of dyslipidemia (including isolated triglyceridemia, isolated cholesterolemia and mixed) was high, with the condition affecting 65.29% of patients in the DR group and 58.93% in the No-DR group.

Another DR risk factor analyzed was the duration of DM2. The importance of this factor to a higher incidence and progression of DR has been previously reported (26,43,44,58). Consistent with these studies, the present results revealed that patients who had a DM2 history of >15 years had an increased risk of DR (OR, 1.993; 95% CI, 1.028–3.863; P=0.0497) (Table II). Conversely, the age factor did not represent a DR risk, which coincided with a previous report analyzing the correlation between age and the severity of the DR (59).

Regarding gender, it was found that males predominated in both the DR and No-DR groups, which demonstrated that gender was not a risk factor for the development of DR (Table II). Others studies have shown an inconsistency when investigating whether gender is a risk factor for the development of DR; while some studies found a higher prevalence of DR in women (51,60), other studies have claimed that the severity of the disease is associated with the male gender (53,59). Furthermore, studies of the prevalence of DR have indicated that the disease occurs in similar proportions among men and women (26,61,62). Further studies are required to determine the causes of this inconsistency in the prevalence of DR according to gender in different populations.

Contradictory results have also been found for hypertension. While there are reports indicating that hypertension is not a risk factor for DR (44,60), a larger number of studies argue for a link between the presence of hypertension and the development of DR (26,53,59). In addition, several studies have reported an association between DR and the systolic, but not diastolic, blood pressure (43,50–52). In the present study, a significant correlation between the blood pressure values and the development of the DR was not found (P>0.05) (Table II); however, the fact that any hypertension in the analyzed patients was already controlled by medical specialists could have affected the present results.

Another widely studied factor is the use of insulin in patients with DM2 to control the disease. Insulin therapy was found to be significantly associated with DR in the patients with DM2 in the present study (P=0.0290) (Table II); however, the OR obtained indicates that insulin therapy is not a risk factor for DR (OR, 0.4026; 95% CI, 0.1793–0.9040). This finding suggests that, in the Mexican population analyzed, treatment with insulin prevented the development of DR in DM2 patients receiving insulin therapy, compared with DM2 patients that did not receive this therapy. The present results differed from studies with Japanese (52) and Iranian (53) patients with DM2, which reported insulin therapy to be a DR risk factor.

Concerning the genetic analysis, PCR-RFLP assays were used to analyze two polymorphisms in intron G of the α2 gene (3,160 A/G and 3,090 T/C) using *Bgl*II and *Nde*I restriction enzymes; these polymorphisms were found to be associated with the presence of DR in the diabetic patients. The results obtained for the genotyping of the α2 gene are the first, to the best of our knowledge, to be made for the Mexican population. The distribution of the *Bgl*II/*Nde*I genotypes, as well as the frequency of the respective alleles, in the DR and No-DR patients was similar (Table IV). With both polymorphisms, the heterozygous genotypes were the most prevalent in the DR and No-DR patients. Statistical analysis revealed no association between the *Bgl*II/*Nde*I genotypes and the development of DR in this patients group (P>0.05) (Table IV).

The fact that this study did not find a significant correlation between the *Bgl*II/*Nde*I genotypes of the α2 gene and DR in the analyzed Mexican population marks an important precedent in this type of study aimed at finding the genes associated with diabetic complications in Mexican population. This finding, together with the absence of the *Bgl*II (3,160 A/A) and *Nde*I (3,090 T/T) genotypes in the analyzed population with DM2 reflects the ethnic differences and genetic heterogeneity among populations. Furthermore, it was observed in the present study that the *Bgl*II/*Nde*I polymorphisms were not in HWE. Deviation from the HWE was noted in both groups (DR and No-DR); the fact that there was deviation from the HWE in the No-DR group indicated that one or more of the model requirements may have been violated. If the possibility of genotyping error is discarded it can be assumed that there are other factors that influenced this outcome and that directly affected the observed allele frequencies. We suggest that the existence of inbreeding in the group analyzed and the insufficient sample size could have been the possible causes of this result, as well as the factors influencing this outcome that directly affected the observed allele frequencies. In the case of inbreeding, although the selection criteria excluded a family relationship between patients, at the level of the population analyzed this phenomenon could be occurring. Furthermore, it was observed that the genotypic frequencies of the *Bgl*II and *Nde*I polymorphisms showed no significant difference in the two study groups (DR and No-DR). Another factor that cannot be ruled out is the sample size, and particularly the group size of the No-DR patients. Given the vast problem of DM2 in the Mexican population and the lack of adequate control of this disease, there were few patients who had a ≥10-year history of DM2 who did not exhibit some degree of DR.

The present results differed from similar studies in Asian and Caucasian populations, where there was a significant correlation between the presence of the *Bgl*II genotype (A/A) and the *Bgl*II (A) allele in the development of DR (3,25). To date, few studies have reported genes associated with DR, and the reported results are inconsistent (63). DM2 is a multigenic and multifactorial disease; therefore the DR depends on the factors driving the DM2 (59,63). Similar results to those described in the present study can be found in the literature, particularly in an analysis conducted on the association between the α2β1 807T allele and the development of DR in French patients with DM2, giving support to the present findings (64).

The polymorphisms in the G intronic region containing the *Bgl*II and *Nde*I recognition sites are used to infer the α2 gene haplotypes, including polymorphisms 807C and 873T of exons 7 and 8, respectively. These polymorphisms have been associated with differences in the platelet α2β1 receptor density (41,65) and several diseases, such as retinal diseases (66,67), colorectal cancer (68) and ischemic stroke risk (69). Based on the HWE results obtained in the present study, and considering that i) the method of Kritzik *et al* (41), which was used in the present study to detect the α2 gene polymorphisms 807C and 873T, is an indirect detection method; and ii) the intronic region, where the recognition sites of *Bgl*II and *Nde*I are present, is the gene region with most susceptibility to variations, we suggest that the intronic *Bgl*II and *Nde*I polymorphic sites in the Mexican population are not linked to exonic 807C and 873T nucleotide polymorphic sites and therefore cannot detect the α2 gene haplotypes reported (41).

In conclusion, the results of the present study indicate a high prevalence of DR that had not been previously described in patients with type 2 diabetes from Northeastern Mexico. No significant association was detected between the frequencies of the genotypes and alleles for the α2 gene polymorphisms *Bgl*II and *Nde*I and the development of DR in the DM2 population. The duration of DM2 ≥15 years was the only risk factor linked with the development of DR.

## Figures and Tables

**Table I.** Baseline characteristics of the type 2 diabetic patients.

Characteristic	No-DR group	DR group	P-value
Age, years	61.6±11.48	58.7±9.51	0.0616
Total cholesterol, mg/dl	223.2±70.83	232.7±71.78	0.2668
Tryglicerides, mg/dl	198.4±93.12	188.0±123.10	0.3189
Glucose, mg/dl	165.5±64.63	168.0±74.11	0.9321
Systolic blood pressure, mmHg	119.3±15.24	123.9±19.04	0.6873
Diastolic blood pressure, mmHg	76.1±10.62	76.9±12.86	0.2384

Data are presented as the mean ± standard deviation. DR, diabetic retinopathy.

**Table II.** Analysis of risk factors for DR in patients with DM2.

Variable	No DR, n (%)	DR, n (%)	P-value
Gender			
Male	35 (62.50)	73 (60.33)	0.8693
Female	21 (37.50)	48 (39.66)	
Age^a^			
<50 years	7 (12.50)	17 (14.28)	0.8181
≥50 years	49 (87.50)	102 (85.71)	
Duration of DM2^a^			
<15 years	36 (65.45)	58 (48.74)	0.0497
≥15 years	19 (34.54)	61 (51.26)	
Hypertension			
Yes	36 (64.29)	80 (66.12)	0.8655
No	20 (35.71)	41 (33.88)	
Cataract			
Yes	18 (32.14)	49 (40.50)	0.3204
No	38 (67.86)	72 (59.50)	
Glaucoma			
Yes	3 (5.36)	14 (11.57)	0.2745
No	53 (94.64)	107 (88.43)	
Kidney disease			
Yes	6 (10.71)	26 (21.49)	0.0957
No	50 (89.29)	95 (78.51)	
Insulin therapy			
Yes	9 (16.07)	39 (32.23)	0.0290
No	47 (83.93)	82 (67.77)	
Dyslipidemia			
Yes	33 (58.93)	79 (65.29)	0.5027
No	23 (41.07)	42 (34.71)	
Glycemia			
Yes	44 (78.57)	89 (73.55)	0.5758
No	12 (21.43)	32 (26.45)	

a Missing data. DR, diabetic retinopathy; DM2, type 2 diabetes mellitus.

**Table III.** Family characteristics associated with DR in the patients with DM2.

Characteristic	No-DR group, n (%)	DR group, n (%)	P-value
DR			
Yes	9 (16.07)	31 (25.62)	0.1802
No	47 (83.93)	90 (74.38)	
DM2			
Yes	35 (62.50)	95 (78.51)	0.0291
No	21 (37.50)	26 (21.49)	
Dyslipidemia			
Yes	23 (41.07)	49 (40.50)	1.0000
No	33 (58.93)	72 (59.50)	
Hypertension			
Yes	33 (58.93)	70 (57.85)	1.0000
No	23 (41.07)	51 (42.15)	
Cardiovascular disease			
Yes	23 (41.07)	42 (34.71)	0.5027
No	33 (58.93)	79 (65.29)	
Kidney disease			
Yes	6 (10.71)	19 (15.70)	0.4884
No	50 (89.29)	102 (84.30)	

DR, diabetic retinopathy; DM2, type 2 diabetes mellitus.

**Table IV.** Genotype and allele frequencies of α2 integrin *Bgl*II and *Nde*I polymorphisms in patients with and without DR.

A, *Bgl*II						

Genotypes and alleles	No-DR group, n (%)	DR group, n (%)	χ^2^	OR	95% CI	P-value
Genotypes						
GG	21 (37.50)	31 (25.60)	2.5895	1.7362	0.87–3.43	0.1076
AG	35 (62.50)	90 (74.40)	2.5895	0.5760	0.29–1.14	0.1076
AA	0 (0)	0 (0)				
Alleles						
G	77 (68.75)	152 (62.80)	1.1794	1.3017	0.81–2.11	0.2775
A	35 (31.25)	90 (37.20)	1.1794	0.7682	0.47–1.23	0.2775

B, *Nde*I						

Genotypes and alleles	No-DR group, n (%)	DR group, n (%)	χ^2^	OR	95% CI	P-value

Genotypes						
CC	2 (3.60)	7 (5.80)	0.3865	0.6047	0.08–2.82	0.5341
TC	54 (96.40)	114 (94.20)	0.3865	1.6536	0.35–11.95	0.5341
TT	0 (0)	0 (0)				
Alleles						
C	58 (51.80)	28 (52.90)	0.0375	0.9567	0.61–1.50	0.8464
T	54 (48.20)	14 (47.10)	0.0375	1.0452	0.66–1.64	0.8464

DR, diabetic retinopathy; OR, odds ratio; CI, confidence interval.

**Table V.** Association of *Bgl*II and *Nde*I genotypes with clinical and biochemical characteristics in patients with DM2.

A, *Bgl*II (risk allele, A; no-risk allele, G)				

	Frequency of AA/AG/GG genotypes, n (%)			
			
Variable	DR patients	No-DR patients	P-value^c^	OR (95% CI)
Cataract	0 (0.0)/44 (65.7)/23 (34.3)	0 (0)/81 (73.6)/29 (26.4)	0.261	0.68 (0.35–1.32)
Duration of DM2 (≥15 years)^a^	0 (0.0)/58 (72.5)/22 (27.5)	0 (0)/76 (80.8)/18 (19.1)	0.192	0.62 (0.31–1.27)
Dyslipidemia^a^	0 (0.0)/56 (77.8)/16 (22.2)	0 (0.0)/69 (65.7)/36 (34.3)	0.084	1.83 (0.92–3.63)
Cardiovascular disease^a^	0 (0.0)/52 (80.0)/13 (20.0)	0 (0.0)/73 (65.2)/39 (34.8)	0.037	2.14 (1.04–4.40)
Hypertension^a^	0 (0.0)/75 (72.8)/28 (27.2)	0 (0.0)/64 (73.6)/10 (26.4)	0.029	2.39 (1.08–5.29)
DM2^a^	0 (0.0)/95 (73.1)/35 (26.9)	0 (0.0)/30 (63.8)/17 (36.2)	0.234	1.54 (0.76–3.13)
B, *Nde*I (risk allele, T; no-risk allele, C)				

	Frequency of TT/TC/CC genotypes, n (%)			
			
Variable	DR patients	No-DR patients	P-value^c^	OR (95% CI)

Cataract	0 (0.0)/62 (92.5)/5 (7.5)	0 (0.0)/106 (96.4)/4 (3.6)	0.262	0.47 (0.12–1.81)
Duration of DM2 (≥15 years)^b^	0 (0.0)/74 (92.5)/6 (7.5)	0 (0.0)/89 (94.7)/5 (5.3)	0.560	0.69 (0.20–2.31)
Dyslipidemia^a^	0 (0.0)/69 (95.8)/3 (4.2)	0 (0.0)/99 (94.3)/6 (5.7)	0.646	1.39 (0.34–5.76)
Cardiovascular disease^a^	0 (0.0)/62 (95.4)/3 (4.6)	0 (0.0)/106 (94.6)/6 (5.4)	0.829	1.17 (0.28–4.84)
Hypertension^a^	0 (0.0)/97 (94.1)/6 (5.8)	0 (0.0)/71 (95.9)/3 (4.0)	0.596	0.68 (0.17–2.82)
DM2^a^	0 (0.0)/124 (95.4)/6 (4.6)	0 (0.0)/44 (93.6)/3 (6.4)	0.637	1.41 (0.34–5.88)

a Family history data

b missing data

c correlation/trend P-value. DM2, type 2 diabetes mellitus; OR, odds ratio; CI, confidence interval.
